# Some Scientific Aspects of Insanity

**Published:** 1891-11-28

**Authors:** 


### Some Scientific Aspects of Insanity.

### IV.—Consciousness and Mind.

Besides being the receptacle of all the in-going sensory currents from the organism the brain performs the additional function of introducing them into consciousness. It is also conscious of every movement of the body. Every in-going current and every out-going current is therefore more or less present in consciousness. When an action is constantly performed, or when a sensory impression is being constantly received by the brain so long as it is not painful we become less and less vividly conscious of it until, as in the act of walking, we forget the motion, and in the presence of sunlight we are oblivious to its rays. On the other hand, every new action is according to its degree of complexity vividly present in consciousness. So is every fresh combination of sensory impressions. It is within the power of an individual to call any act, however common, at any moment into consciousness. And we therefore assume that the higher centres of the brain are continually receiving impressions, the result of the most recent and the most vividly conscious acts, and the most common and least vividly conscious acts. Therefore, consciousness may be said to be the sum of three separate classes of nerve currents: 1st, of sensory impressions received from the outside world; 2nd, of sensory impressions received from every part of the body in the performance of its vital functions; 3rd, of impressions of varying intensity received from the nervous system itself on the liberation of any out-going nerve current. The fusion of these three elements with associated brain changes which they themselves are constantly arousing and which are called ideas, recollections, and associations, is called a mental state. The mental state is continuously changing with the character of the in-going currents and the vitality of the organism; but there is no interruption in the connection of one mental state with that which immediately precedes it and follows it. Mind and consciousness are one—that is to say, it is our mind that is conscious of those actions of our nervous system. It is quite unnecessary here to enter into the various theories which are assigned to account for the connection between mind and matter. Suffice it to say that every action of the nervous system is, as it were, mirrored in mind. Our nervous systems stand in relation to mind in the same way as a man does to his shadow or to his reflection in a mirror. It has for many hundred years been a disputed question among philosophers whether mind is acted upon through matter, that is, through the nervous system, or whether it is that mind itself only acts upon the nervous system, much as an organist strikes the keys of his instrument. However that may be, we cannot dissociate the brain and mind. When the brain is healthy, the mental manifestations are healthy also. When the brain is diseased there is disordered mind. Just in the same way as when a happy man looks in a mirror you see his reflection to be bright and pleasant; and when a sorrowful man stands in the same position his image is sad and downcast. Sanity, therefore, requires healthy brain substance; insanity is always accompanied by a diseased brain condition.

The grey matter of the brain composed of cells is a mechanism for storing up and liberating nervous energy. The white matter composed of fibres is a system of wires for the transmission to and from the various parts of the body of nervous energy. Now, if there happens to be an injury or a disease of the grey matter there will be an interference with its property of liberation and storing. If, for instance, there is an injury of the motor part of the brain, say in the centre for movements of the right arm, there will be paralysis of voluntary motion of that arm. In the same way, if there be a lesion of white matter there will be an interference in the transmission of nervous energy along the fibres in which the injury occurs. If there be an injury of the fibres in any part of their course conveying motor impressions from the motor area of the brain to the right arm there will be paralysis of the arm. Again if the gray matter of the brain which receives impressions from any one part of the body be diseased the impressions will not be perceived. Thus if the middle convolution of the temporal lobe be diseased the sense of hearing on that side will be affected. An injury of fibres conveying impressions from any part of the body will effectually prevent the transmission of the impression; if, for instance, the fibres of the auditory nerves were destroyed the impression on the ear made by waves of sound would not be conveyed to the brain. We therefore see that any injury to the nervous system, or destruction of any part of it, is of very serious import. As a rule, the changes that take place in the nervous system in insanity are extremely minute, and subtle, and are only observable under high powers of the microscope. They consist of degenerations and wastings of the small cells of which the grey matter of the cortex is so largely composed; but there is another and important change to be noted, and it is the destruction of those minute fibres by which one cell is joined to another.

The grosser injuries which are observed in cases of paralysis of the various parts of the body are but types of the more subtle changes that occur in insanity. Insanity has been defined as a failure on the part of the individual to adapt himself to his surroundings. Now, when a man has an injury of the grey matter in the motor cortex which presides over the movements of one of his legs, or when he has an injury in the white fibres that descend from that cortex, either in the brain or in the spinal cord, the man has paralysis of that leg. He will fail, therefore, to adapt himself to all the exigencies that may occur in his surroundings. In the same way, but acting upon nervous tissue more highly complex and evolved, and which, instead of presiding over motion directly, presides over the formation of memories, emotions, and conduct, a lesion of nerve-cells or nerve-fibres occurring in the highest parts of the brain will cause an interference with the highest and most important functions of the nervous system. The power of judging correctly is, perhaps, the highest, as it is the most complex, duty of the brain, and it requires the perfectly harmonious action of the highest centres of the brain to perform this function. Just as in disordered feeling there may be disease of the cells of the brain set apart for the reception of the sensation, or of the nerve-fibres that convey the sensation to that part, so in disordered judging power there is either disease of the most special and higher brain-cells or of the fibres going to these cells. We discern that a man is paralysed by the way he walks, that he is blind by his inability to see obstacles which he stumbles over, that he is insane by his conduct and behaviour, which indicate defective judging power and consequent erroneous conduct.

